# Time of Energy Intake: Association with Weight Status, Diet Quality, and Sociodemographic Characteristics in Brazil

**DOI:** 10.3390/ijerph21111403

**Published:** 2024-10-24

**Authors:** Paulo Rogério Melo Rodrigues, Luana Silva Monteiro, Thaís Meirelles de Vasconcelos, Iuna Arruda Alves, Edna Massae Yokoo, Rosely Sichieri, Rosangela Alves Pereira

**Affiliations:** 1 Faculdade de Nutrição, Federal University of Mato Grosso, Avenida Fernando Corrêa da Costa, 2367, Cuiabá 79070-900, Brazil; 2 Instituto de Alimentação e Nutrição, Federal University of Rio de Janeiro, Avenida Aluizio da Silva Gomes, 50, Macaé 21941-617, Brazil; luananutrir@gmail.com; 3 Programa de Pós-Graduação em Saúde Coletiva, State University of Ceará, Avenida Dr. Silas Munguba, 1700, Fortaleza 60714-903, Brazil; thaismvasconcelos@gmail.com; 4 Instituto de Nutrição Josué de Castro, Federal University of Rio de Janeiro, Avenida Carlos Chagas Filho, 373, Rio de Janeiro 50740-580, Brazil; iunaarrudanut@gmail.com (I.A.A.); roapereira@gmail.com (R.A.P.); 5 Instituto de Saúde Coletiva, Fluminense Federal University, Travessa Marquês de Paraná, 303/3 Andar, Niterói 24020-141, Brazil; eyokoo@gmail.com; 6 Instituto de Medicina Social, State University of Rio de Janeiro, Rua São Francisco Xavier, 524, Pavilhão João Lyra Filho, 7° Andar, Rio de Janeiro 20950-000, Brazil; rosely.sichieri@gmail.com

**Keywords:** chrono-nutrition, time of energy intake, obesity, dietary intake, risk factors, nutrition survey

## Abstract

This study aimed to estimate the association of time of energy intake with weight status, diet quality, and sociodemographic characteristics in Brazil. This cross-sectional study used data from a nationally representative survey with 44.744 individuals (≥10 years old). Food consumption was assessed by 24 h recall. The evening/morning energy intake ratio was calculated, standardized, and categorized in tertiles. The association between the evening/morning energy intake ratio and weight status was estimated using polynomial logistic regression models, and differences across diet quality and sociodemographic categories were estimated considering the non-overlapping 95% confidence intervals. Men, adolescents, adults, and individuals in the higher income level had greater evening energy intake. Those with a higher evening-to-morning energy intake ratio were 15% more likely to be obese (OR = 1.15; 95% CI = 1.02 to 1.28), 21% less likely to be underweight (OR = 0.79; 95% CI = 0.64 to 0.98), and reported greater total energy, protein, and lipid intake, as well as higher consumption of low-quality diet markers. Higher evening energy intake relative to morning intake was associated with obesity, low-quality diet markers, and sociodemographic characteristics. The characterization of the time of energy intake can be useful for tailoring and targeting diet promotion actions and for controlling the obesity epidemic.

## 1. Introduction

Obesity represents an important burden on population health and is considered a major public health problem, given its great magnitude and serious health repercussions. The determination of obesity is multifactorial, involving genetic causes, psychosocial factors, and obesogenic environments, which are conditioned by structural factors that lead to reduced access to healthy foods and hinder physical activity. In Brazil, data from national surveys conducted between 1974 and 2019 showed a reduction in the prevalence of undernutrition and an increase in overweight and obesity; the latter, in 2019, reached 20,3% of the population and is estimated to affect three out of 10 Brazilian adults by 2030 [1,2,3].

In Brazil, like other modern societies [4], traditional foods are being replaced by energy-dense diets high in sugar, sodium, and fats and low in fiber, vitamins, and minerals [5,6]. Louzada et al. [6] analyzed the variation of ultra-processed food consumption in Brazil between 2008–2009 and 2017–2018 and observed that, in 2017–2018, these foods accounted for 19.7% of energy intake. Moreover, the authors pointed out that, between the two periods, the groups with the greatest increase in ultra-processed food consumption were individuals in the lowest education level (+1.18 percentage points-pp), men (+1.59 pp), black people (+2.04 pp), those living in rural areas (+2.43 pp) and in the north (+2.95 pp) and northeast (+3.11 pp) regions, and those classified in the lowest income quintile (+3.54 pp) [6].

Recent social, economic, and cultural modifications have resulted in changes in eating habits, including escalating unhealthy eating patterns, increased number of daily consumption occasions, increased portion sizes, and changes in eating times, for example, more frequent late-night eating [7,8]. The latter are aspects related to the circadian rhythm [9,10], which regulates several physiological responses, including metabolism, that can be impaired when there is a mismatch between physiological circadian timing and food intake timing [11,12]. Therefore, timing of food/nutrient intake can be considered a modifiable lifestyle factor for the circadian physiology involved in weight gain and metabolism [13].

In this context, chrono-nutrition is a field of nutritional epidemiology that addresses eating timing, frequency, and regularity [12] and the complex relationship of those three dimensions with circadian rhythms and metabolic health [10]. The circadian rhythms represent the innate 24 h cycles in human behavior, physiology, and metabolism, which are influenced by light exposure [10]. In addition to the sleep–wake cycle, the circadian rhythm regulates several functions in the body, such as body temperature, hormonal regulation, liver and kidney metabolism, and heart rate [12,14]. This cycle is regulated by internal and external synchronizers, also called zeitgebers [15]. Among the zeitgebers, there are photic synchronizers, such as light, and non-photic synchronizers, like food/nutrition, temperature, stress, and exercise. Food consumption has been referred to as a zeitgeber [16] because it can regulate various peripheral body clocks and metabolic rhythms, especially in the liver and intestine [17,18].

Thus, the association between the timing of energy intake and obesity has been explored in several studies. In particular, late evening eating has been associated with excessive weight gain. In a study with 239 US individuals between 21 and 69 years old, Wang et al. [19] showed that greater intake of energy in the evening, compared to the morning or afternoon, was associated with a higher risk of overweight/obese (OR = 2.00; 95% CI = 1.03–3.89). Likewise, among 3610 Swedish men and women, late-night eating compared with no late-night eating was associated with obesity (OR = 1.62; 95% CI, 1.10–2.39) [20]. Xiao et al. [21], with 872 US middle- to older-aged adults, observed that a higher percent of total daily energy intake consumed within 2 h before bedtime was associated with higher odds of being overweight/obese (OR = 1.82; 95% CI = 1.07–3.08), especially among individuals reporting being a later chronotype (OR = 4.94; 95% CI = 1.61–15.14). The chronotype refers to the individual circadian preferences, related to the preferred time to sleep and to be active: in the morning or in the evening, with some people being flexible regarding the times they choose to be active or resting [14,22].

Late evening eating has also been associated with inadequate diet quality. Among 3.304 Japanese women between 18 and 20 years old, Sato-Mito et al. [23] observed that evening-type subjects (higher food consumption in the evening) had significantly lower potassium, calcium, magnesium, iron, zinc, vitamin A, thiamine, riboflavin, pyridoxine, folate, and vitamin D intakes compared with the morning-type individuals. In Brazil, Teixeira et al. [24], studying 721 >18-year-old undergraduate students using a morningness–eveningness questionnaire, observed that the evening type was associated with skipping breakfast and greater total fat and total energy intake per day.

We hypothesized that higher evening energy intake relative to morning intake is associated with obesity and poor diet quality. Furthermore, we sought to explore the sociodemographic characteristics related to evening food consumption in order to identify the population groups that should be targeted by actions to promote healthy eating. Thus, this study aimed to analyze data from a nationally representative survey in Brazil to estimate the association of the timing of energy intake with weight status, diet quality, and sociodemographic characteristics.

## 2. Methods

The data utilized in this cross-sectional study were derived from the Brazilian National Dietary Survey (NDS), which examined a subsample of the household participants in the 2017–2018 National Household Budget Surveys (HBS) conducted by the Brazilian Institute of Geography and Statistics (acronym in Portuguese: IBGE). The complex sampling plan applied in the HBS is predicated upon a master sample comprising the selection of census sectors (primary sample units) that have been stratified based on geographic location, urban or rural situation, household income levels, and households (secondary sample units). The NDS subsample (*n* = 20.112 households; 34.7% of original sample) was obtained through simple random sampling [25]. In the 2017–2018 NDS, data on food consumption were collected for 46.164 individuals ≥10 years old, and in the present analysis, pregnant and lactating women were excluded (*n* = 1.420); thus, data from 44.744 Brazilians were analyzed.

### 2.1. Dietary Intake Assessment

The 24 h recall (24hR) method was applied to evaluate food consumption on two non-consecutive days selected within a one-week span. Participants were asked to recall all food and beverages (including water) consumed on the day preceding the interview. The interviews were based on the multiple-pass method [26] using a computational tool particularly designed for this assessment. For every food and beverage item, participants were asked to provide details regarding the quantity, occasion, place, and time of consumption. Additionally, at the end of the interview, the participants were informed if that corresponded to a typical or atypical day of food consumption. Energy and selected nutrient intake were estimated using the Brazilian Food Composition Table (https://www.tbca.net.br/ (accessed on 10 September 2024)) [27]. The foods reported in the 24 h recall were classified into 28 groups based on their nutritional attributes (see Supplementary chart).

In this analysis, the ratio of evening to morning energy intake was estimated for each individual [28]. Morning energy intake was calculated as the sum of energy intake reported between 6:00 am and 11:00 am. Evening energy intake was estimated as the sum of energy intake reported between 6:00 pm and 00:00 am. These time intervals were selected based on prevailing patterns of morning and evening meal consumption observed for the Brazilian population [29]. Subsequently, the ratio of evening to morning energy intake was estimated, standardized (to obtain a distribution mean equal to zero and standard deviation equal to one), and stratified into tertiles. Therefore, those classified in the first tertile had lower energy intake in the evening than in the morning; conversely, individuals classified in the third tertile had greater energy intake in the evening compared to the morning.

### 2.2. Weight Status and Sociodemographic Variables

The explanatory variables analyzed in this study were sex (male/female), age group (adolescents: 10–19 years old; adults: 20–59 years old; and elderly: ≥60 years old), and monthly per capita family income (calculated from the sum of total family income divided by the number of individuals in the family and categorized into multiples of the Brazilian official monthly minimum wage effective in the middle of the data collection in <0.5 minimum wages per capita (MWPC), 0.5 to 1.0 MWPC, 1.0 to 2.0 MWPC, and ≥2.0 MWPC).

Weight and height were informed by the participants, and weight status was assessed using the body mass index (BMI, kg/m^2^). Adolescents [30], adults, and elderly [31] were classified according to the criteria proposed by the World Health Organization. Individuals were classified as underweight, normal weight, overweight, or obesity.

### 2.3. Statistical Analyses

The proportion of individuals in each tertile of the evening-to-morning energy intake ratio was estimated for the categories of sex, age group, income, and weight status, along with their respective 95% confidence intervals (95% CI), and differences in the proportions across the categories were assessed by non-overlapping 95% confidence intervals (95% CI).

The association between the evening/morning energy intake ratio (independent variable; the reference category was the first tertile) and weight status (dependent variable; the reference category was normal weight) was estimated using a polynomial logistic regression model adjusted by sex, age, per capita family income, and total energy intake, estimating the odds ratios and respective 95% CIs.

Means (and 95% CIs) of energy and selected nutrient intake and the proportions (%; 95% CIs) of food group reports were estimated across the tertiles of the evening/morning energy intake ratio. Differences in means and proportions across the tertiles were assessed by non-overlapping 95% CIs.

The analysis incorporated sample weights and accounted for the study design’s effect using the Complex Sample module of the Statistical Package for the Social Sciences (SPSS), version 19 (IBM SPSS Statistics).

## 3. Results

In the present study, 50.7% were female, 63.9% were adults, and 31.9% had per capita family income between 1 and 2 minimum wages; 46.0% were classified as having normal weight, 36.0% were classified as overweight; and 15.5% were classified as obesity. The standardized evening/morning energy intake ratio in the first tertile was <−0.19; in the second tertile, this ratio varied between −0.19 and 0.37; while in the third tertile, the ratio was ≥0.37 (Table 1). Mean, morning energy intake (6 am–11 am) was 440.5 kcal (25% of daily energy intake), afternoon energy intake (11 am–6 pm) was 776.5 kcal (44% of daily energy intake), and evening energy intake (6 pm–0 am) was 546.7 kcal (31% of daily energy intake).

A greater proportion of women (31.9%) compared to men (28.2%) were classified in the first tertile of the evening/morning energy intake ratio; the same was observed for the elderly (37.6%) compared to adults (28.5%) and adolescents (28.1%) and for individuals in the lowest income level (33.0%) compared to the highest (27.0%). Conversely, a lower proportion of women (33.5%) were classified in the third tertile of the evening/morning energy intake ratio compared to men (38.6%); the same was observed for elderly (26.8%) compared to adults (38.0%) and adolescents (38.2%) and for low-income individuals (32.4%) compared to those with higher incomes (38.2%) (Table 1).

**Figure 1:**
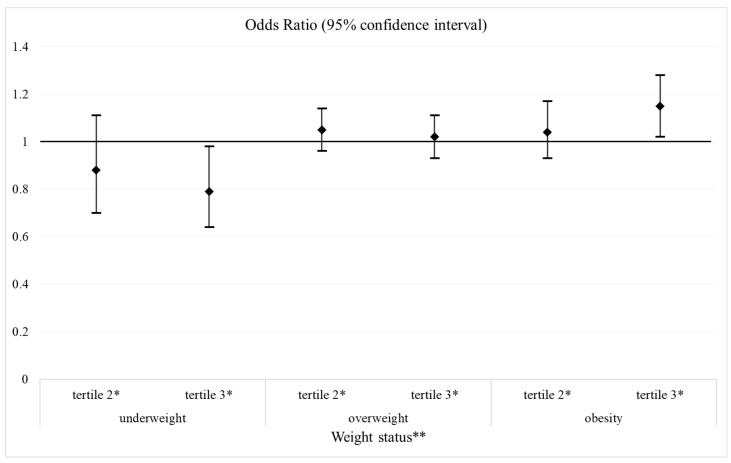
Association (odds ratio and 95% confidence interval) between the evening/morning energy intake ratio and weight status, Brazilian National Dietary Survey, 2017–2018. * reference category: tertile 1. ** reference category: normal weight. Estimated using polynomial logistic regression model, having as dependent variable the weight status and the evening/morning energy intake ratio as the independent variable and adjusted by sex, age, total energy intake, and per capita family income (estimated from the total household income divided by the number of household members) in multiples of the country official minimum wage at the time of the study, corresponding to USD 297.00.

Compared to those in the first tertile of the evening/morning energy intake ratio, individuals in the third tertile had a greater chance of obesity (OR = 1.15; 95% CI = 1.02; 1.28, *p* = 0.018) and lower chance of underweight (OR = 0.79; 95% CI = 0.64; 0.98, *p* = 0.029) (Figure 1).

Individuals classified in the first tertile of the evening/morning energy intake ratio, compared with those in the third tertile, had lower mean energy (1669 vs. 1797 kcal), protein (17.4 vs. 19.3% of total energy intake-TEI), lipid intake (29.0 vs. 30.2% of TEI), fiber intake (12.8 vs. 13.7 g/1000 kcal), and sodium intake (1436.2 vs. 1465.8 mg/1000 kcal) and higher carbohydrate (55.7 vs. 52.4% of TEI), calcium intake (259.0 vs. 244.3 mg/1000 kcal), and added sugar intake (10.3 vs. 9.3% of TEI) (Table 2).

Compared to individuals in the third tertile, there was a greater proportion of individuals classified in the first tertile of evening/morning energy intake ratio reporting the consumption of coffee and tea (84.3 vs. 76.6%), sugar (66.3 vs. 60.0%), breads (56.2 vs. 40.4%), solid fats (42.7 vs. 28.4%), fruits (32.1 vs. 25.9%), milk and dairy (25.3 vs. 20.0%), corn and corn-based dishes (15.1 vs. 11.0%), and whole grains (7.1 vs. 5.2%) and lower proportion of those reporting the consumption of red meats (50.3 vs. 55.1%), fast foods (22.0 vs. 27.2%), sugar-sweetened beverages (16.3 vs. 24.1%), pasta and pasta-based dishes (19.9 vs. 23.4%), vegetables oils (12.0 vs. 15.2%), sweets and desserts (11.9 vs. 14.5%), and sauces (4.1 vs. 6.9%) (Table 3).

**Figure 2:**
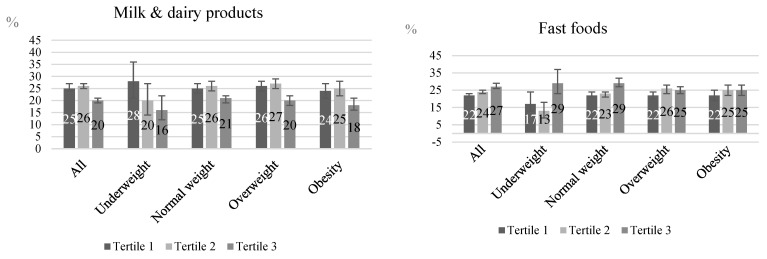

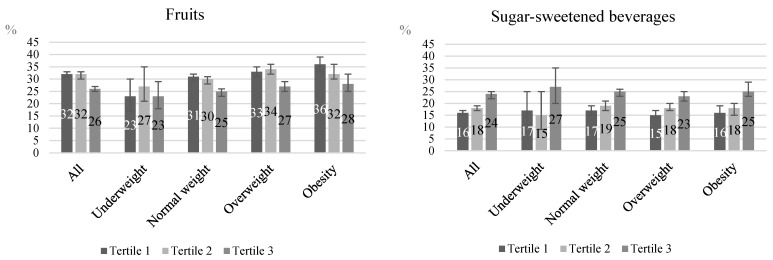
Intake of milk and dairy products, fast foods, fruits, and sugar-sweetened beverages according to the tertile of evening/morning energy intake ratio and weight status. Brazilian National Dietary Survey, 2017–2018.

Furthermore, differences in the report of food group consumption across the tertiles of the evening/morning energy intake ratio were independent of the weight status, as exemplified in Figure 2 for milk and dairy, fast foods, fruits, and sugar-sweetened beverages.

## 4. Discussion

In the 2017–2018 Brazilian Dietary Survey, our results are consistent with the hypothesis that higher evening energy intake relative to morning intake is associated with obesity; increased energy, protein, lipid, fiber, and sodium intake; and greater consumption of food groups that are characteristic of a low-quality diet. Moreover, it was inversely associated with the odds of being underweight. Men, adolescents, adults, and individuals in the highest income category presented a greater evening/morning energy intake ratio than their counterparts.

Similar to the findings of the present study, the International Study on Macro/Micronutrients and Blood Pressure (INTERMAP) demonstrated that British and American adults with lower evening/morning energy intake ratio had lower BMI and higher dietary nutrient density. In opposition, individuals who consumed the majority of their energy intake in the evening had a lower intake of fruits and vegetables, as well as lower dietary nutrient density [28]. Correspondingly, Gontijo et al. [32], in a cross-sectional study with 100 Brazilian pregnant women in the first gestational trimester, observed lower diet quality and reduced consumption of fruits and grains among those who were prone to nighttime consumption. Lower intake of fruits and vegetables alongside higher consumption of alcoholic beverages, sugar-sweetened beverages, caffeinated drinks, and greater energy intake from fat was observed among individuals with a nocturnal feeding habit in literature reviews [9,33,34].

This study is in line with findings on the association between increased nighttime consumption and adverse health outcomes and poorer diet quality. Kanbay et al. [35], in a literature review, found that nighttime eating habits, characterized by high caloric intake later in the evening, have been associated with hyperlipidemia, hypertriglyceridemia, hyperglycemia, weight gain, elevated blood pressure, obesity, metabolic syndrome, and atherosclerosis. Likewise, other studies indicate that the later the last meal of the day was, the higher the risk of metabolic and cardiovascular outcomes. Findings from prospective cohort studies involving shift workers [36] and from a cross-sectional study with workers from oil and gas installations in the United Kingdom [37] found that individuals who predominantly consumed a large proportion of their energy during the night had higher risk of developing coronary heart disease and type 2 diabetes. Wirth et al. [38] analyzed data from the National Health and Nutrition Examination Survey (NHANES 2005–2016) and observed that eating late was associated with higher levels of glycosylated hemoglobin, insulin, and C-reactive protein. In the NutriNet-Santé, a large prospective cohort study, having the last meal late in the day significantly contributes to increasing the risk of adverse cardiovascular outcomes, especially among women [39].

The observed associations of the greater evening intake relative to morning intake with critical metabolic outcomes may be attributed to an interaction between metabolism and the circadian system [8]. Evidence suggests that the effects of the circadian system on metabolism may contribute to metabolic dysfunctions [10,11], thereby predisposing individuals to metabolic diseases such as obesity [9,40].

In a randomized, open-label, crossover study involving individuals with type 2 diabetes, Jakubowicz et al. [41] observed that the distribution of calories consumed at breakfast or dinner influenced the daily rhythm of postprandial glycemic and insulin levels. In another study, these authors also reported that omission or delayed breakfast triggers discordance between endogenous circadian clock rhythms and the feeding/fasting cycle, and this phenomenon is associated with an increased incidence of obesity and type 2 diabetes [40].

Peters et al. [8] noted, in a review study, that mistimed food intake, such as late or nighttime consumption, can lead to desynchronization of the internal circadian clock and increase the risk for obesity and associated metabolic disturbances, for example, type 2 diabetes. The authors highlight that the chronotype may play an important role in meal timing preference. Chronotypes can range from morning to evening preferences and may be influenced by the circadian system, genetics, the light–dark cycle, and environmental factors [8]. Furthermore, chronotype can be described as a complex phenotype representing individual preference [42] and is characterized by the preferred sleep–wake timing and timing for carrying out daily activities, including eating habits [43]. Therefore, recognizing the chronotypes may be useful for understanding variations in the timing of food intake [8,9]. Nevertheless, Dashti et al. [44] emphasize that diverse factors may determine the timing of eating, including environmental, cultural, behavioral, and physiological factors, for example, work routine like shift work, irregular part-time work, or long work hours [8]. The intensified urbanization process has been associated with extended wake time and, consequently, fewer sleeping hours and late eating habits; thus, Tiuganji et al. [45] suggest that eating habits may vary according to the place of living. In this perspective, life conditions, culture, work schedules, and social obligations may also affect meal timing [8,46].

In the present study, weight status was classified based on self-reported weight and height measurements, which could be considered a limitation. However, previous research has demonstrated that self-reported weight and height measurements are reliable in Brazilian studies among adolescents [47,48] as well as among adults and the elderly [49]. The underreporting of energy intake is common in the collection by 24 h recall. However, a validation study with the 2008–2009 Brazilian National Dietary Survey data revealed that the 24 h recall showed less underestimation of energy intake than the food record [50]. The use of a single day to assess food consumption could also be considered a limitation. However, a single day of 24 h recall is sufficient for estimating the mean intake of populations [51], and data from the first day of 24 h recall tend to be of higher quality compared to subsequent days [52].

Additionally, different approaches and measures used to assess eating and meal timing can impair the comparability of studies and their interpretation. Similar to the present study, the ratio of evening/morning energy intake was used in the INTERMAP study [28]. In the NHANES 2005–2016 [38] and NutriNet-Santé cohort [39], the authors considered the timing of the last meal of the day. Differences between the energy density of dinner and breakfast [41], as well as fasting until noon due to omitted or delayed breakfast [40], have also been evaluated. The cross-sectional design of the present study prevents drawing a causal relationship between the timing of energy intake and health outcomes. However, there is consistency in the results when observing associations between late food consumption or higher evening energy intake relative to morning intake with adverse health and diet quality outcomes.

This study provides robust estimates from large and nationally representative data of the Brazilian ≥10-year-old population, which stands out as a strength of the present analysis. Additionally, the method used to obtain food consumption data can also be considered a strength of this study, as the 24 h recall allows for detailed documentation of food consumption, including information on timing and eating occasions [53]. In the 2017–2018 NDS, various strategies were adopted to minimize misreporting, including obtaining food consumption data with the assistance of computational resources and designing the 24 h recall interview based on the multiple-pass method [26]. Furthermore, studies evaluating the timing of energy intake in Brazil and its associations with weight status, diet quality, and sociodemographic characteristics are scarce in the country.

Therefore, the findings of the present study represent an innovative approach toward acknowledging the importance of a balanced distribution of food consumption throughout the day, with a greater emphasis on morning intake relative to evening intake. Eating timing is rarely addressed in dietary guidelines; however, evidence suggests that this topic should be incorporated into these guidelines since it is another line of action to promote healthy eating and reduce obesity and related diseases. In addition, the recognition of eating timing may be relevant for the establishment of food nutritional interventions based on food and nutrition education, as well as for tailoring and targeting healthy eating promotion actions.

## 5. Conclusions

In Brazil, greater evening energy intake was associated with a higher chance of obesity chance, lower odds of being underweight, and low diet quality and was more frequent in men, adolescents, adults, and high-income individuals. Therefore, characterizing the time of energy intake can be valuable for tailoring and targeting healthy eating promotion initiatives and assisting efforts to minimize the obesity epidemic.

## Figures and Tables

**Table 1:** Characterization of the sample and distribution according to the tertile of the ratio of evening/morning energy intake, Brazilian National Dietary Survey, 2017–2018.

		Tertile of Evening/Morning Energy Intake Ratio [% (95% CI)]		
	Total	1 (<−0.19)	2 (−0.19; 0.37)	3 (>0.37)
Total		30.1 (29.3; 30.9)	33.9 (33.1; 34.7)	36.0 (35.1; 36.9)
Sex				
Men	49.3	28.2 (27.2; 29.1)	33.3 (32.2; 34.3)	38.6 (37.4; 39.7)
Women	50.7	31.9 (30.9; 33.0)	34.5 (33.5; 35.6)	33.5 (32.5; 34.6)
Age group				
Adolescents	17.9	28.1 (26.6; 29.6)	33.7 (31.9; 35.5)	38.2 (36.3; 40.2)
Adults	63.9	28.5 (27.6; 29.4)	33.5 (32.5; 34.4)	38.0 (36.9; 39.1)
Elderly	18.1	37.6 (36.0; 39.2)	35.6 (34.0; 37.3)	26.8 (25.1; 28.4)
Per capita family income				
½	16.6	33.0 (31.1; 34.9)	34.6 (32.5; 36.8)	32.4 (30.3; 34.5)
½ to 1	24.2	30.9 (29.4; 32.4)	34.6 (33.2; 36.0)	34.5 (33.1; 36.0)
1 to 2	31.9	30.6 (29.2; 32.0)	32.2 (31.0; 33.6)	37.2 (35.5; 38.9)
>2	27.3	27.0 (25.6; 28.5)	34.8 (33.0; 36.6)	38.2 (36.3; 40.0)
Weight status				
Underweight	2.5	35.0 (30.9; 39.3)	33.4 (29.1; 38.0)	31.6 (27.7; 35.8)
Normal weight	46.0	30.0 (28.9; 31.1)	33.7 (32.6; 34.8)	36.4 (35.2; 37.6)
Overweight	36.0	30.2 (29.0; 31.4)	34.6 (33.3; 35.9)	35.2 (33.9; 36.6)
Obesity	15.5	29.5 (27.7; 31.3)	33.1 (31.4; 34.9)	37.4 (35.4; 39.4)

95% CI = 95% confidence intervals. Per capita family income (estimated from the total household income divided by the number of household members), in multiples of the country’s official minimum wage at the time of the study, corresponding to USD 297.00.

**Table 2:** Means of energy, macronutrients, added sugar, and sodium intake according to the tertile of evening/morning energy intake ratio, Brazilian National Dietary Survey, 2017–2018.

	Tertile of Evening/Morning Energy Intake Ratio		
	1	2	3
	[Mean (95% CI)] *		
Energy (kcal)	1669 (1648; 1690)	1765 (1743; 1787)	1797 (1770; 1824)
Protein (% of total energy intake)	17.4 (17.2; 17.6)	18.4 (18.1; 18.6)	19.3 (19.1; 19.5)
Carbohydrate (% of total energy intake)	55.7 (55.4; 56.0)	54.6 (54.3; 55.0)	52.4 (52.0; 52.7)
Lipids (% of total energy intake)	29.0 (28.7; 29.2)	29.3 (29.1; 29.5)	30.2 (29.9; 30.5)
Fiber (g/1000 kcal)	12.8 (12.6; 13.0)	13.2 (13.0; 13.3)	13.7 (13.5; 13.9)
Calcium (mg/1000 kcal)	259.0 (254.1; 263.8)	256.9 (252.3; 261.5)	244.3 (239.1; 249.4)
Iron (mg/1000 kcal)	6.4 (6.3; 6.5)	6.5 (6.4; 6.6)	6.3 (6.2; 6.3)
Vitamin C (mg/1000 kcal)	71.0 (67.6; 74.4)	68.2 (65.5; 71.0)	69.2 (65.7; 72.7)
Saturated fat (% of total energy intake)	9.3 (9.2; 9.4)	9.3 (9.2; 9.4)	9.3 (9.2; 9.4)
Added sugar (% of total energy intake)	10.3 (10.1; 10.5)	9.6 (9.4; 9.9)	9.3 (9.1; 9.5)
Sodium (mg/1000 kcal)	1436.2 (1423.1; 1449.3)	1462.7 (1448.8; 1476.6)	1465.8 (1448.9; 1482.7)

* Adjusted for sex, age, and income.

**Table 3:** Proportion of individuals reporting the consumption of food groups according to the tertile of evening/morning energy intake ratio, Brazilian National Dietary Survey, 2017–2018.

	Ratio of Evening/Morning Energy Intake		
Food Groups	1	2	3
	[% (95% CI)]		
Coffee and tea	84.3 (83.3; 85.3)	82.9 (81.6; 84.1)	76.6 (75.1; 78.1)
Rice and rice dishes	76.7 (75.2; 78.1)	78.6 (77.0; 80.1)	77.7 (75.8; 79.5)
Beans and bean dishes	75.5 (74.2; 76.7)	76.8 (75.3; 78.2)	74.3 (72.6; 75.9)
Sugar	66.3 (64.8; 67.7)	66.7 (65.1; 68.2)	60.0 (58.4; 61.6)
Breads	56.2 (54.8; 57.6)	58.6 (57.2; 60.1)	40.4 (38.8; 41.9)
Red meats	50.3 (48.8; 51.7)	54.7 (53.1; 56.2)	55.1 (53.5; 56.7)
Vegetables	43.3 (41.9; 44.7)	47.3 (45.7; 48.9)	44.6 (42.9; 46.3)
Solid fats	42.7 (41.2; 44.2)	44.1 (42.5; 45.7)	28.4 (27.0; 29.8)
Poultry and poultry dishes	34.0 (32.6; 35.4)	35.4 (33.9; 37.0)	35.1 (33.5; 36.7)
Fruit juices	32.2 (30.9; 33.5)	34.0 (32.4; 35.5)	33.4 (31.8; 35.1)
Fruits	32.1 (30.8; 33.4)	31.6 (30.2; 33.0)	25.9 (24.6; 27.3)
Roots and tubers	30.1 (28.7; 31.5)	30.5 (29.1; 32.0)	30.9 (29.4; 32.4)
Cookies and crackers	27.8 (26.6; 29.1)	26.9 (25.5; 28.3)	25.8 (24.5; 27.1)
Milk and dairy	25.3 (24.1; 26.6)	25.8 (24.5; 27.2)	20.0 (18.8; 21.2)
Fast-foods	22.0 (20.8; 23.2)	23.8 (22.6; 25.1)	27.2 (25.6; 28.8)
Pasta and pasta-based dishes	19.9 (18.8; 21.0)	21.2 (20.0; 22.5)	23.4 (22.0; 24.8)
Sugar-sweetened beverages	16.3 (15.1; 17.4)	18.4 (17.2; 19.6)	24.1 (22.8; 25.5)
Corn and corn-based dishes	15.1 (14.1; 16.1)	13.0 (12.1; 14.0)	11.0 (10.1; 11.9)
Eggs	14.6 (13.6; 15.6)	15.4 (14.4; 16.5)	13.2 (12.1; 14.3)
Cakes	14.5 (13.6; 15.5)	13.8 (12.9; 14.8)	12.9 (11.9; 13.9)
Vegetable oils	12.0 (11.0; 13.1)	15.5 (14.4; 16.8)	15.2 (13.9; 16.6)
Sweets and desserts	11.9 (11.0; 12.8)	12.1 (11.2; 13.1)	14.5 (13.4; 15.6)
Processed meats	11.2 (10.3; 12.2)	12.4 (11.3; 13.7)	11.9 (11.0; 12.9)
Non-caloric sweetener	8.8 (8.0; 9.7)	9.5 (8.8; 10.4)	7.3 (6.5; 8.1)
Fish and seafood	8.0 (7.3; 8.9)	7.7 (7.0; 8.4)	8.4 (7.6; 9.3)
Whole grains	7.1 (6.3; 7.9)	7.2 (6.5; 8.0)	5.2 (4.5; 6.0)
Milk-based processed beverages	7.0 (6.4; 7.7)	8.0 (7.2; 8.9)	6.8 (6.0; 7.6)
Sauces	4.1 (3.5; 4.7)	5.2 (4.5; 6.0)	6.9 (6.1; 7.7)

## Data Availability

The data that support the findings of this study are openly available in the Brazilian Institute of Geography and Statistics at https://www.ibge.gov.br/estatisticas/sociais/saude/24786-pesquisa-de-orcamentos-familiares-2.html?=&t=micro data (accessed on 10 September 2024).

## Supplementary Materials

The following supporting information can be downloaded at https://www.mdpi.com/article/10.3390/ijerph21111403/s1. Supplementary chart. Food groups. Brazilian National Dietary Survey, 2017–2018.
