# Supplementary material for: Time of Energy Intake: Association with Weight Status, Diet Quality, and Sociodemographic Characteristics in Brazil
Source: Int J Environ Res Public Health. 2024 Oct 24;21(11):1403. doi: 10.3390/ijerph21111403 (PMC11593663; doi:10.3390/ijerph21111403)
Supplement: Supplementary file 1 [file ijerph-21-01403-s001.zip › ijerph-3231066-supplementary.pdf]

Supplementary chart. Food groups. Brazilian National Dietary Survey, 2017–2018

**Food groups**	**Food items**
**Cookies & crackers**	Crackers (regular, light), cookies, sandwich cookies
**Fruits**	Non specified fruit, açaí berry, acerola, apple, avocado, banana, cashew, fruit salad, grapes, guava, jabuticaba (Plinia cauliflora), jackfruit, lemon, lime, mango, melon, orange, papaya, peach, persimmon, pineapple, plantain, plum, raisins, strawberry, sugar cane, tangerine, watermelon
**Sugar**	Table sugar, brown sugar, raw sugar, honey, liquid and solid sugarcane molasses
**Non-caloric sweetener**	Non caloric sweeteners
**Fruit juices**	Non specified fruit juice, fresh squeezed juice of: acerola, cupuaçu, grape, guava, hog plum (*Spondias mombin*), orange, passion fruit, pineapple, strawberry, sugarcane,green leaves, coconut water
**Fast-foods**	Calzone, pizza, hot dogs, hamburger, assorted sandwiches, ham & cheese sandwich, chips, baked or fried dumplings, cheese rolls, savory pies
**Sweets & desserts**	Candies, lollipops, bubble gum, cereal bars, bonbons, chocolate bar, coconut candy, fruit preserves, milk-based desserts, meringue, peanut-based sweets, jelly, jam, ice cream, popsicle, chocolate powder, honey cake, condensed milk and condensed milk-based confectionery, caramel candy
**Coffee & tea**	Infusions prepared from yerba mate *(Ilex paraguariensis)*, tea, instant coffee, cappuccino, coffee, coffee with milk
**Milk-based processed beverages**	Fermented milk, flavored milk, yogurt, soy milk-based drinks, whey-based processed drinks
**Sugar-sweetened beverages**	Soft drinks, sodas, energy drinks, processed fruit-based drinks
**Breads**	Non specified bread, bread with butter/margarine, buns and rolls, sliced loaf, French bread, Australian bread, homemade bread, corn bread, toast
**Vegetable oils**	Non specified vegetable oil, olive oil, soy oil, coconut oil
**Solid fats**	Butter, margarine, lard, bacon, pork skin, cream
**Milk & dairy products**	Milk, goat's milk, powdered milk, smoothies, curdled milk, Kefir, porridge (cornmeal, oats, or other), assorted cheeses (white and yellow)
**Cakes**	Cake, cake with icing, sweet rolls, panettone, donuts, sweet tarts and pies, brownie, cupcake
**Whole grains**	Brown rice, granola, whole grain pasta, whole wheat biscuits, whole wheat bread
**Corn &corn-based dishes**	Ear of corn, polenta, cornmeal mush, popcorn
**Sauces**	Mustard, shoyu, tartar sauce, ketchup, salad dressing, mayonnaise (regular, light)
**Processed meats**	Ham, turkey breast, bologna, salami, sun-dried beef, jerked beef, *pâté*, sausages
**Red meats**	Beef, pork, organ meats, and red meat dishes.
**Poultry & poultry dishes**	Chicken, quail, duck, turkey, poultry dishes.
**Fish & seafood**	Fish, shellfish, seafood, shrimp, cod, octopus, sashimi.
**Eggs**	Eggs, quail eggs, and omelette.
**Vegetables**	Asparagus, beets, broccoli, cabbage, carrot, chayote, cauliflower cucumber, eggplant, garlic, gilo (*Solanum gilo*), green beans, heart of palm, leafy vegetables, leeks, mushroom, okra, olives, onion, bell pepper, pumpkin, radish, tomato, snow peas, zucchini
**Roots &tubers**	Cassava, potato, sweet potato, yam, arracacha, taro, tapioca, cassava flour
**Beans & bean dishes**	Beans, lentils, chickpeas, soy, beans dishes
**Rice & rice dishes**	Rice, risotto, and other rice dishes
**Pasta and pasta-based dishes**	Spaghetti, lasagne, capeletti, cannelloni, gnocchi, yakissoba, pancake, instant noodles, meat or vegetable broth, onion, cheese or vegetable chowder, vegetable or bean soup
